# Incidence of preoperative high blood pressure in cataract surgery among hypertensive and normotensive patients

**DOI:** 10.4103/0301-4738.71679

**Published:** 2010

**Authors:** Rodrigo Pessoa Cavalcanti Lira, Maurício Abujamra Nascimento, Carlos Eduardo Leite Arieta, Luis Eduardo Mateus Duarte, Fabio Endo Hirata, Wilson Nadruz Junior

**Affiliations:** Department of Ophthalmology, Universidade Estadual de Campinas - UNICAMP, Campinas, Brazil

**Keywords:** Cataract, high blood pressure, systemic diseases

## Abstract

Incidence of preoperative rise in blood pressure (BP) in cataract surgery among hypertensive and normotensive patients.

**Objective:**

To study the incidence of preoperative rise in BP in cataract surgery among normotensive individuals and hypertensive patients with historic good BP control in a population without other major chronic diseases.

**Settings:**

Ophthalmology Service of a University Hospital.

**Materials and Methods:**

A prospective study with 822 patients older than 40 years of age, with cataract surgery indication, and without major chronic diseases other than hypertension. The patients were divided in two groups: hypertensive and normotensive. Preoperative data, physical exams and medical adverse events were recorded in an evaluation questionnaire.

**Results:**

The sample included 427 normotensive (52%) and 395 hypertensive patients (48%). The two groups had similar proportions of operations that were cancelled and not subsequently rescheduled, 2% (eight patients) in each group. The incidence of preoperative rise in BP was 3.7% in the normotensive group and 10.9% in the hypertensive group (*P* < 0.001).

**Conclusion:**

Hypertensive patients with historic good BP control and without other major co-morbidities present a larger incidence of preoperative rise in BP than normotensive individuals in cataract surgery.

High blood pressure (BP) is one of the most common diseases in the world and it is clear that its prevalence increases with aging. Cataract is one of the most frequently performed surgeries and most patients are over 60 years old, exactly the individuals most affected by hypertension.[1–4]

Two-thirds of the patients who undergo cataract surgery have at least one chronic disease. Hypertension is the most prevalent of those, present in 50% of these individuals.[3–5]

Medical non-ocular complications in cataract surgery occur in 2–15%. The most frequent are high BP, bronchospasm and cardiac arrhythmia, accounting for more than 90% of the adverse events. Among those, high BP is the most prevalent.[3,4]

The purpose of this research was to study the incidence of preoperative rise in BP in cataract surgery among normotensive individuals and hypertensive patients with historic good BP control in a population without other major chronic diseases.

## Materials and Methods

### Patients and medical procedures

The study was carried out at the State University of Campinas, Brazil, between June 2008 and May 2009. The medical center’s ethics committee approved the study protocol. All patients provided written informed consent before enrolment in the study.

Patients below 40 years old, with diabetic, heart, vascular, thyroid, brain or kidney diseases, besides any ocular or systemic disease that required use of chronic medication were excluded from the study.

The patients who agreed to participate in the study were divided into two groups: normotensive individuals and hypertensive patients. The criteria for the diagnosis of hypertension were those defined by the Seventh Report of the Joint National Committee on Prevention, Detection, Evaluation, and Treatment of High BP (VII JNC). The measurements of the BP were performed with a mercury manometer, following the JNC guidelines. All patients were evaluated one month before surgery by a cardiologist to assure that BP had a historic good control (systolic blood pressure – [SBP] < 140 mmHg and diastolic blood pressure, [DBP] < 90 mmHg).[6]

Data were collected by means of a medical history form, completed by the physician at the preanesthetic medical examination. Preoperative high BP was recorded on a standardized form by a member of the nursing staff. All patients received routine preoperative medication, which consisted of diazepam 5 mg administered orally 1 h before surgery. The BP was measured 30 min after medication. The definition of preoperative rise in BP was systolic ≥ 180 mm Hg or diastolic ≥ 110 mm Hg with necessity of new antihypertensive treatment or change of the actual medication.

A sample size of 720 patients (360 per group) was planned. Assuming a preoperative high BP rate of 9% in the hypertensive group, this sample size provided a 90% probability of detecting a difference as small as 6% in the normotensive group. Results of these analyses were considered as statistically significant when the *P* values were < 0.05. We used Epi Info 2000 computer software (United States Center for Disease Control and Prevention, Atlanta, Georgia, United States of America) for the statistical analyses. For categorical variables, chi-square (Yates) tests were used; for continuous variables, one-way analysis of variance (ANOVA) was used.

## Results

We enrolled 822 patients scheduled to undergo cataract surgery. The sample included 427 normotensive (52%) and 395 hypertensive patients (48%). The two groups had similar proportions of operations that were cancelled and not subsequently rescheduled, 2% (eight patients) in each group. The two groups were well balanced in terms of age, with a mean age of 66 years (SD = 11 years) in the normotensive group and of 66 years (SD = 12 years) in the hypertensive group (*P* < 0.734). The groups were also well balanced with respect to gender with 224 males (52.5%) in the normotensive and 216 males (54.7%) in the hypertensive group (*P* < 0.466).

**Figure 1:**
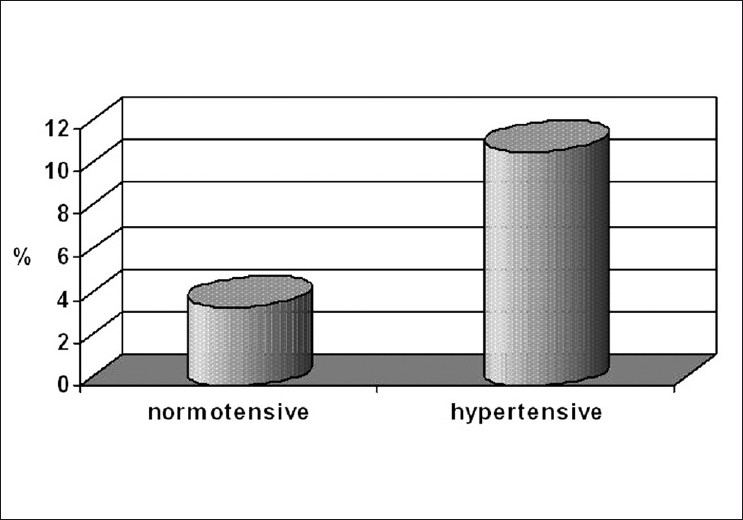
Incidence of preoperative rise in blood pressure in cataract surgery among hypertensive and normotensive patients

The incidence of preoperative rise in BP was 3.7% (16 patients) in the normotensive group and 10.9% (43 patients) in the hypertensive group (*P* < 0.001) Fig. 1.

## Discussion

The results of this study confirm that hypertensive patients, even those with a history of good BP control are at increased risk for rise in BP in the perioperative period. However, no additional problems with the patients with higher BP were reported during or after the surgery.

Mild to moderate hypertension (Stage 1 or Stage 2) affects patients with SBP < 180 mmHg and DBP < 110 mmHg. The evidence suggests that these patients do not appear to be at increased operative risk for cardiovascular adverse outcomes. For patients with severe hypertension (Stage 3, SBP th ≥ 180 mmHg and DBP th ≥ 110 mmHg), BP should be controlled prior to proceeding to elective surgery. The BP target should be less than 140/90 mmHg in patients with uncomplicated hypertension, less than 130/85 mmHg in those with diabetes, and less than 125/75 mmHg in those with renal disease. Emerging data support a target BP of less than 150/80 mmHg in patients more than 80 years old.[6–9]

Patients commonly present for preoperative evaluation to the primary care physician, consultant, surgeon, or anesthesiologist with either diagnosed or undiagnosed hypertension. Even if a patient carries the diagnosis of hypertension and takes antihypertensive therapy, the hypertension may be poorly controlled. In the United States, approximately 50% of patients who are aware of their hypertension are either not treated or inadequately treated with pharmacological therapy. Patients should continue taking preoperative antihypertensive medications throughout the entire perioperative period. Various treatment strategies include dietary modifications, regular aerobic exercise, weight loss, and pharmacotherapy. Patients with hypertension are at a higher risk for labile BP and for hypertensive emergencies during surgery. Intravenous esmolol, labetalol, nitroprusside, or nitroglycerin may be used for acute episodes of hypertension, whereas calcium channel blockers or angiotensin-converting enzyme inhibitors may be used in less acute situations. The decision to delay surgery for long-term stabilization of BP, versus acutely correcting the hypertension, must balance the potential benefits and the urgency of the surgical procedure.[1,10,11]

Even in mild to moderate hypertensive patients, studies showed that the risk is not nil. It was demonstrated that untreated or unsatisfactorily controlled hypertensive patients, developed episodes of perioperative rise in BP more severe than normotensive or well-controlled hypertensive patients, besides the greater risk of cardiac arrhythmias or myocardial ischemia. Systemic arterial hypertension and high systolic BP in preoperative admission were associated with a greater incidence of silent myocardial ischemia.[12,13]

As patients with diseases other than hypertension were excluded from the study, we cannot generalize our results. It is important to emphasize that the focus of our study was a relatively healthy population undergoing a low-risk procedure. However, that fact is an advantage of this research, since the sample population did not present other diseases, which could act as confounding factors in data analysis.[14]

The preoperative evaluation is a unique opportunity to identify patients with hypertension and to evaluate them for adequate control of condition. These results reinforce the importance of preoperative evaluation of hypertension in cataract patients.

## Conclusion

Hypertensive patients with historic good BP control and without other major comorbidities present a larger incidence of preoperative rise in BP than normotensive individuals in cataract surgery.
